# Association of ovine gammaherpesvirus 2 with an outbreak of acute respiratory disease in dairy cattle

**DOI:** 10.1038/s41598-023-30133-w

**Published:** 2023-04-06

**Authors:** Selwyn Arlington Headley, Alais Maria Dall Agnol, José Antonio Bessegato, Ana Paula Souza Frucchi, Érika Fernandes Lopes Maturana, Rafael Vince Rodrigues, Ana Aparecida Correa Xavier, Alice Fernandes Alfieri, Amauri Alcindo Alfieri

**Affiliations:** 1 Laboratory of Animal Pathology, Department of Preventive Veterinary Medicine, Universidade Estadual de Londrina, Rodovia Celso Garcia Cid, PR 445 Km 380, Campus Universitário, PO Box 10.011, Paraná, 86057-970 Brazil; 2 National Institute of Science and Technology for Dairy Production Chain (INCT – LEITE), Department of Preventive Veterinary Medicine, Universidade Estadual de Londrina, Paraná, Brazil; 3 Multi‑User Animal Health Laboratory, Tissue Processing Unit, Department of Preventive Veterinary Medicine, Universidade Estadual de Londrina, Londrina, Paraná Brazil; 4 Laboratory of Animal Virology, Department of Preventive Veterinary Medicine, Universidade Estadual de Londrina, Paraná, Brazil; 5 Consulting Veterinarian, Herd Bovinos - Consultoria Pecuária, Dois Vizinhos, Paraná, Brazil; 6 Multi‑User Animal Health Laboratory, Molecular Biology Unit, Department of Preventive Veterinary Medicine, Universidade Estadual de Londrina, Londrina, Paraná Brazil

**Keywords:** Infectious-disease diagnostics, Pathogens, Herpes virus, Microbiology, Molecular biology, Diseases, Pathogenesis

## Abstract

This study investigated the cause of an outbreak of an acute respiratory disease syndrome followed by episodes of diarrhea in a dairy cattle herd from Southern Brazil. Deep nasal swabs (DNS) from asymptomatic calves, calves with pulmonary discomfort, and diarrheic calves after episodes of respiratory distress were used in molecular assays designed to detect the principal pathogens associated with bovine respiratory disease (BRD). Fecal samples were used for the molecular detection of bovine enteric disease agents. Pulmonary tissues from three calves and a cow that died were evaluated by molecular assays to identify 11 agents associated with the development of BRD. The intestinal and pulmonary fragments of one calf and the cow revealed atrophic enteritis and interstitial pneumonia by histopathology, respectively. Immunohistochemistry (IHC) identified intralesional antigens of a malignant catarrhal fever virus, genus *Macavirus*, within epithelial cells of the lungs and intestines. Molecular assays amplified ovine gammaherpesvirus 2 (OvGHV2) from most of the DNS, and the pulmonary and intestinal fragments from the animals that died, confirming that the *Macavirus* identified by IHC was OvGHV2. Concomitant pulmonary infections of OvGHV2 with bovine gammaherpesvirus 6 and bovine coronavirus were identified. Additionally, bovine viral diarrhea virus 1b and Aichivirus B were detected in the fecal samples. These findings demonstrated that OvGHV2, a *Macavirus*, was the disease agent most frequently (81.2%; 13/16) associated with singular pulmonary infections during this outbreak of BRD, suggesting that this virus may be another potential agent of respiratory disease of cattle.

## Introduction

Ovine gammaherpesvirus 2 (OvGHV2) and bovine gammaherpesvirus 6 (BoGHV6) are members of the *Macavirus* genus, subfamily *Gammaherpesvirinae*, family *Herpesviridae*^1^. OvGHV2 is known for the development of several, well-defined, clinical manifestations of sheep-associated malignant catarrhal fever (SA-MCF) in susceptible mammalian hosts worldwide^2–4^. Furthermore, members of the *Macavirus* genus associated with the development of MCF in mammalian hosts are referred to as MCF virus (MCFV), since they share the 15A epitope^5,6^. Additionally, we have developed an immunohistochemical (IHC) assay that uses the 15A monoclonal antibody (MAb-15A) to detect intralesional antigens of MCFV in tissues of ruminants^7^. This antibody detects the 15A epitope present in all recognized MCFV^8^ and has been used effectively in several serological investigations associated with molecular testing and histopathology^9–11^.

Some of the well-known clinical manifestations of SA-MCF include the head and eye^4,12^, alimentary^13–15^, neurological^7,14^, and cutaneous^5,15^ forms. Alternatively, the role of BoGHV6, that was initially isolated in cows with lymphoma^16^, and previously referred to as bovine lymphotropic virus, in the development of disease processes in ruminants is poorly understood and controversial^17^. This is probably because studies did not demonstrate any relationship between the occurrence of BoGHV6 and the development of diseases in ruminants from Europe^18^ and the USA^19^. However, BoGHV6 was associated with lymphoproliferative diseases^20,21^, myocarditis^22^, pneumonia^17^, enteritis^17^, as well as several reproductive disease conditions including endometritis^23,24^, metritis^25,26^, and abortions^22,27^.

The bovine respiratory disease (BRD) complex is a multietiological disease entity that is associated with several infectious disease agents and sudden changes to climatic and environmental conditions and/or management practices. The traditional agents of BRD include bacterial pathogens such as *Mannheimia haemolytica*, *Pasteurella multocida*, *Histophilus somni*, and *Mycoplasma bovis*^28–30^, as well as viral pathogens, such as bovine viral diarrhea virus (BVDV), bovine respiratory syncytial virus (BRSV), bovine alphaherpesvirus 1 (BoAHV1), bovine parainfluenza virus-3 (BPIV-3), and bovine coronavirus (BoCV)^29–31^. We had proposed that OvGHV2 may be another potential disease agent of BRD^2^, and have since identified this virus^7^, or at least a MCFV^32–35^, in association with the development of pulmonary diseases in cattle. Furthermore, since we have associated the occurrence of BoGHV6 with pneumonia in buffalos^17^, it seem likely that these two members of *Macavirus* genus may be potential agents of pulmonary disease of ruminants. However, in most of the previous descriptions of pulmonary lesions associated with *Macavirus*, typical clinical manifestations of pulmonary discomfort were not observed. Accordingly, this study describes the association of OvGHV2 in dairy cattle with clinical manifestations of pulmonary disease.

## Results

### Clinical observations

The calves evaluated during this study were between 1 and 60 days-of age; the age of two calves were unknown (Table 1). Most diarrheic calves (71.4%; 5/7) were more than 2-weeks-old, while calves with respiratory impairment were less than 7-days-of-age. Calf #6 demonstrated the clinical manifestations of a pulmonary syndrome that was characterized by nasal and ocular secretions, dyspnea, hyperthermia, and cough; tissues from this animal were received for pathological and molecular evaluations. Animal #8 was a 4-year-old, cow that recently gave birth to a calf and died after recovering from signs of pulmonary discomfort. The consulting veterinarian reported that this cow (animal #8) was apparently normal after parturition, but suddenly developed muscle tremors, uncoordinated gait, and died within 24 h of the onset of clinical manifestations.

**Table 1:** Molecular and immunohistochemical findings observed in asymptomatic calves and in cattle with respiratory and enteric manifestations.

Animal#	Age (days)	Clinical manifestation	Types of samples		Results					Type of respiratory infection
			Diarrheic	Respiratory	Molecular detection^1^			MCFV IHC		
					BoGHV6	OVGHV2	BCoV	Lung	Intestine	
1	60	Asymptomatic	Normal feces ^b^	Deep nasal swab	−ve	+ve	−ve	NA	NA	Singular
2	55	Asymptomatic	Normal feces	Deep nasal swab	−ve	+ve	−ve	NA	NA	Singular
3	NP	Respiratory	NS	Deep nasal swab	+ve	+ve	−ve	NA	NA	Dual
4	NP	Respiratory	NS	Deep nasal swab	+ve	+ve	−ve	NA	NA	Dual
5	1	Respiratory	NS	Pulmonary tissue	+ve	−ve ^2^	−ve	NS	NS	Singular
6^a^	2	Respiratory	Normal feces ^b,c^	Pulmonary tissue	−ve	+ve ^2^	−ve	+ve	+ve	Singular
7	7	Respiratory	NS	Pulmonary tissue	−ve	+ve ^2^	−ve	NS	NS	Singular
8^a^	4 years	Respiratory	NS	Pulmonary tissue	−ve	+ve ^2^,*	−ve	+ve	+ve	Singular
9	40	Respiratory	Normal feces ^b^	Deep nasal swab	−ve	+ve	−ve	NA	NA	Singular
10	30	Diarrhea	Liquid feces ^b^	Deep nasal swab	−ve	+ve	+ve *	NA	NA	Dual
11	21	Diarrhea	Feces soft	Deep nasal swab	−ve	+ve	−ve	NA	NA	Singular
12	21	Diarrhea	Soft feces	Deep nasal swab	−ve	+ve	−ve	NA	NA	Singular
13	15	Diarrhea	Soft feces	Deep nasal swab	−ve	+ve	−ve	NA	NA	Singular
14	14	Diarrhea	Soft feces	Deep nasal swab	−ve	+ve	−ve	NA	NA	Singular
15	5	Diarrhea	Soft feces ^c^	Deep nasal swab	−ve	+ve *	−ve	NA	NA	Singular
16	2–3	Diarrhea	Soft feces	Deep nasal swab	−ve	+ve	−ve	NA	NA	Singular

*Sequenced samples.

^a^Animals that died of pulmonary distress with tissues submitted for routine histopathologic evaluation and fecal samples obtained during gross evaluation.

^b^Fecal samples infected with BVDV1b.

^c^Fecal samples infected with Aichivirus. −ve, negative; + ve positive, *NP* not provided, *NS* not submitted, *NA* not applicable.

^1^Nucleic acids of BVDV, BoAHV1, BPIV-3, BRSV, *P. multocida*, *H. somni*, *M. haemolytica*, and *M. bovis* were not amplified from the deep nasal swabs or pulmonary tissues.

^2^OvGHV2 DNA was amplified from the intestine of these animals with atrophic enteritis.

### Gross, histopathologic, histochemical, and immunohistochemical findings

**Figure 1:**
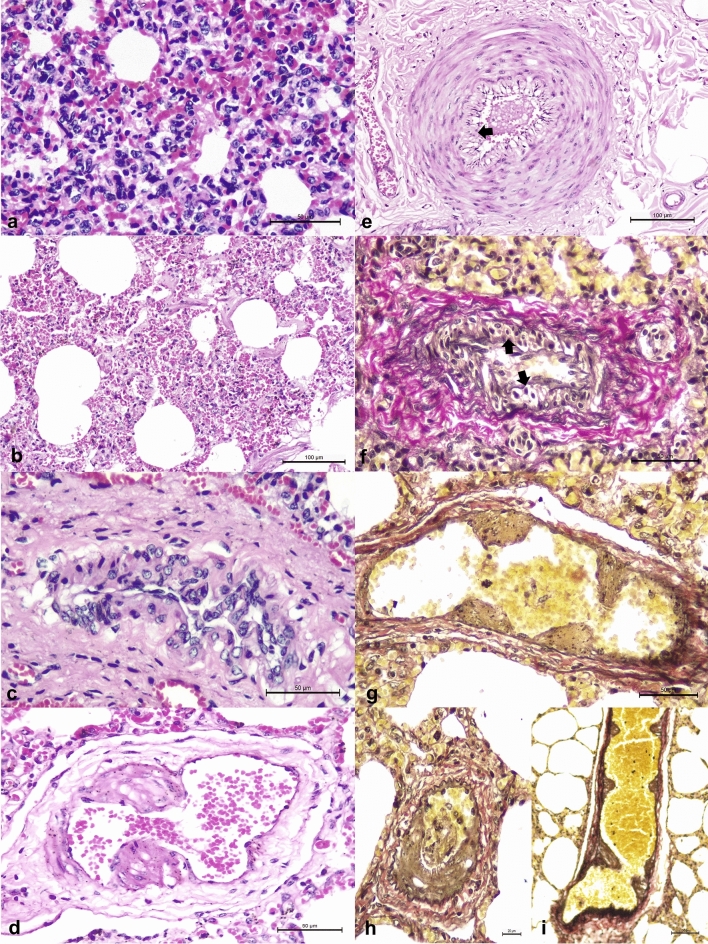
Histopathological and histochemical findings observed in cattle during an outbreak of respiratory disease induced by OvGHV2. Observe interstitial pneumonia in the calf (**a**) and cow (**b**), proliferating vascular lesions in the calf (**c**) and the cow (**d**) and marked endothelial degeneration (arrow) in the cow and calf (**e**,**f**). Observe the proliferative vascular lesions (**g**,**h**), with evidence of partial luminal obliteration (**i**). Hematoxylin and eosin stain (**a**–**e**); the Verhoeff-Van Gieson histochemical method (**f**–**i**). Bar; (**a**,**c**,**d**,**f**,**g**) 50 µm; (**b**,**e**) 100 µm; (**h**,**i**) 20 µm.

The tissues (lung and intestine) received from the calf (animal #6) for histopathologic evaluations did not present any significant gross alteration. While the lungs of the cow were severely congested; no gross abnormalities were observed in the intestinal tissue. Histopathologic evaluation of the lungs of the calf revealed severe, diffused, lymphoplasmacytic interstitial pneumonia, identified by the thickening of alveolar walls due to the severe accumulations of lymphoplasmacytic inflammatory infiltrate^36^ and the proliferation of type II pneumocytes, with proliferative vascular lesions (PVL), and congestion (Fig. 1A). The histologic lesions observed in the cow (animal # 8) consisted of interstitial pneumonia due to the thickening of alveolar septum by alveolar hyalinization and congestion^36^ with marked PVL, pulmonary congestion, and edema (Fig. 1B). In the lungs of both the calf and cow, suppurative and/or fibrinous bronchopneumonia was not observed. The PVLs observed in these two animals were characterized by endothelial vacuolization/degeneration, proliferation of media and/or intima with consequent luminal obliteration, and transmural vascular degeneration (Fig. 1C–F), being more severe in the cow relative to the calf. Histopathology of the intestinal fragments of both animals revealed moderate lymphoplasmacytic atrophic enteritis with PVL. Furthermore, PVL were more occlusive in the cow (#8) relative to the calf (#6) when pulmonary tissues were evaluated with the VVG histochemical stain (Fig. 1G–I).

**Figure 2:**
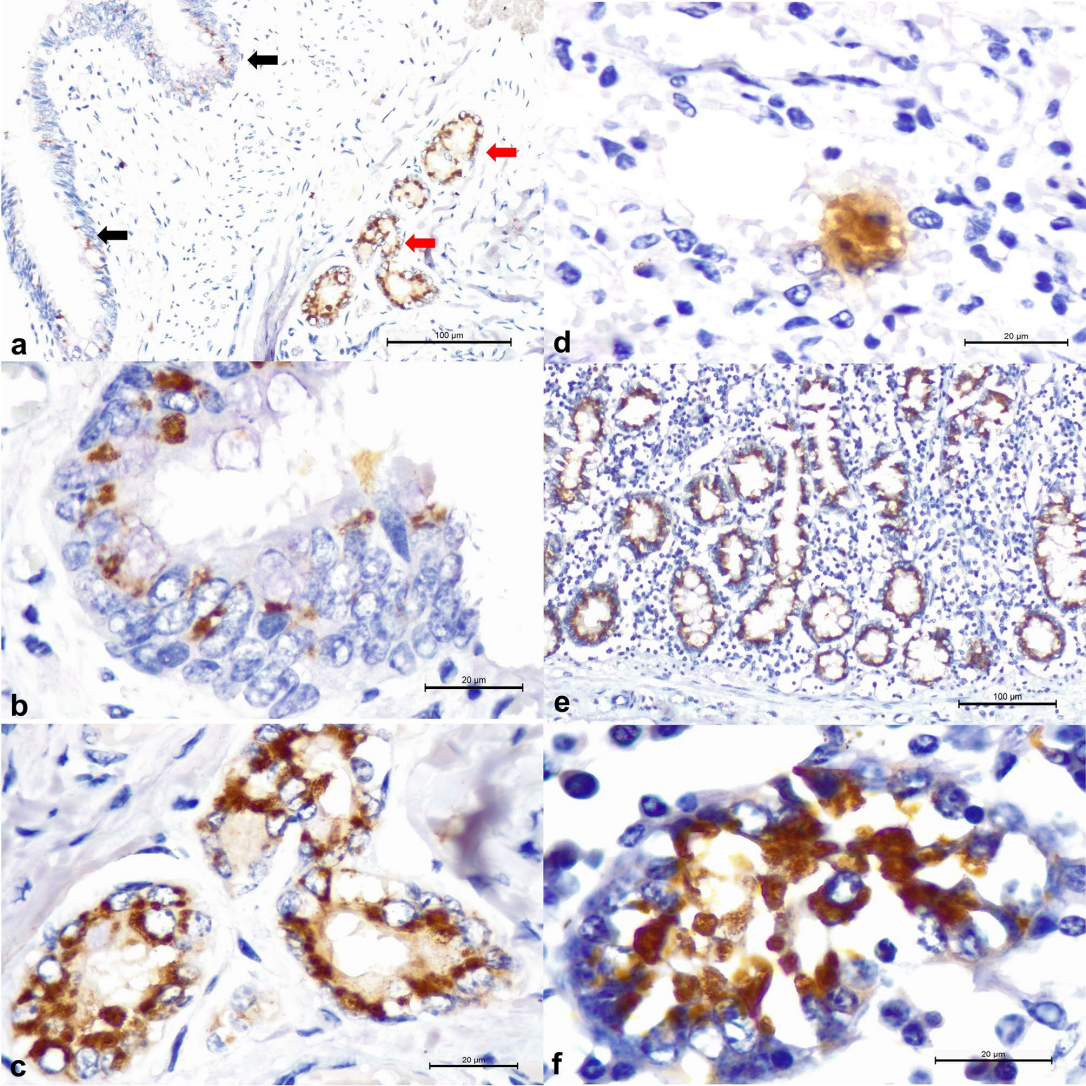
Immunohistochemical identification of MCFV antigens in the lungs and intestines of cattle infected with OvGHV2. There is positive immunoreactivity to MCFV antigens within the epithelial cells of the bronchus (black arrows) and the peribronchial glands (red arrows) of the calf with interstitial pneumonia (**A**); closer view of the intracytoplasmic accumulations of antigens of MCFV within epithelial cells of the bronchus (**B**), peribronchial glands (**C**), and a pneumocyte (**D**). Observe positive intracytoplasmic immunoreactivity to MCFV antigens within epithelial cells of intestinal crypts (**E**), which can be better appreciated at a closer view (**F**). Immunoperoxidase counterstained with Hematoxylin. Bars (**A**,**E**) 100 µm; (**B**–**D**,**F**) 20 µm.

Immunohistochemistry identified intralesional antigens of a MCFV predominantly within the bronchial epithelium and parabronchial glands, with patchy, positive immunoreactivity within pneumocytes of both animals with interstitial pneumonia (Fig. 2A–D). Additionally, there was positive immunoreactivity for MCFV antigens within the epithelial cells of the intestinal crypts of both animals with lymphoplasmacytic atrophic enteritis, with immunopositivity being more widespread and severe in the cow relative to the calf (Fig. 2E–F). Furthermore, the antigens of BoAHV1, BRSV, BVDV, and *M. bovis* were not identified within the FFPE tissues evaluated. Controls for the MCFV IHC assay and the normal distribution of elastin with the VVG stain are provided (Supplementary Fig. 1).

### Molecular confirmation of the association of OvGHV2, BoGHV6, and BCoV with respiratory disease

The molecular assays amplified the respective genes of OvGHV2, BoGHV6, and BCoV from the samples evaluated. The nt sequences for the OvGHV2 strains herein identified, were named OvGHV2/BRA/UEL/PR-447/22 and OvGHV2/BRA/UEL/PR-450/22, are deposited in GenBank (accessions # OP121120 and OP121121), and demonstrated 97.8 and 98.7% sequence identity, respectively, with the prototype strain (BJ1035) of OvGHV2 (Supplementary Table 1). Similarly, the BoGHV6 and BCoV strains herein identified were named BoGHV6/BRA/UEL/PR-450/202 and BCoV/BRA/PR-447-320/2022-N, respectively, are deposited in GenBank (BoGHV6, OP121119; BCoV, OP121122) and had 100% and 99.2% nt sequence identity, respectively, with reference strains for BoGHV6 and BCoV.

### Molecular detection of pathogens associated with respiratory and enteric diseases of cattle

Nucleic acids of OvGHV2 were amplified from most (93.3%; 14/15) of the DNS and pulmonary samples received from the cattle at this farm, except for the pulmonary fragment of a 1-day-old calf (#5) with clinical manifestations of respiratory distress (Table 1). However, this calf was only infected by BoGHV6. Additionally, two calves (# 3 and 4) with clinical signs of the acute pulmonary syndrome were simultaneously infected by OvGHV2 and BoGHV6, while a 1-month-old calf (#10) was concomitantly infected by OvGHV2 and BCoV, being the only infection by BCoV identified during this outbreak. In summary, during this outbreak of respiratory disease in dairy cattle, there were singular infections (*n* = 13) due to OvGHV2, with dual (simultaneous) infections associated with OvGHV2 and BoGHV6 (*n* = 2), and OvGHV2 and BoCV (*n* = 1). Furthermore, the nucleic acids of BVDV, BoAHV1, BPIV-3, BRSV, *P. multocida*, *H. somni*, *M. haemolytica*, and *M. bovis* were not amplified from the DNS and/or pulmonary tissues evaluated during this investigation.

In addition, OvGHV2 DNA was amplified from the intestinal fragments of the three calves and the cow (#5–9) submitted for molecular investigation. Moreover, BVDV1b was amplified from the fecal samples of four calves (#1, 6, 9, and 10), while Aichivirus was detected in the fecal samples of two calves (#6 and 15), resulting in dual infections by BVDV1b and Aichivirus in the fecal sample of calf #6. Alternatively, the nucleic acids of BCoV, BRV, and OvGHV2 were not amplified from the fecal samples received from the 11 calves submitted for molecular investigation.

## Discussion

During this investigation, dairy cattle with an initial acute pulmonary syndrome developed diarrhea after approximately 2 weeks of respiratory distress. Histopathologic evaluation of the lungs and intestines of two animals from this farm that died during this outbreak revealed interstitial pneumonia and atrophic enteritis, respectively. Furthermore, IHC assays identified intralesional antigens of a MCFV from the affected organs which were confirmed to be OvGHV2 by molecular characterization. In addition, molecular testing confirmed the participation of OvGHV2 from the DNS and the lungs of most of the animals with this pulmonary syndrome, with similar molecular detection being obtained from the intestinal fragments of the calves with enteric disease. Moreover, BoGHV6 and BCoV were the only other pathogens amplified from the DNS and/or the pulmonary fragments of cattle with pulmonary distress, while agents frequently associated with BRD, such as BVDV, BoAHV1, BPIV-3, BRSV, *P. multocida*, *H. somni*, *M. haemolytica*, and *M. bovis* were not detected, suggesting that these pathogens were not related with the development of the respiratory outbreak at this dairy herd at the time of sampling. Consequently, these findings confirmed that OvGHV2 was the principal agent associated with the outbreak of the acute pulmonary syndrome at this farm and represent the first study to suggest and demonstrate a direct association between infection by OvGHV2 and the development of BRD. Furthermore, these results are in accordance with previous studies by our group that have shown the participation of a *Macavirus*, most likely OvGHV2, in pulmonary disease syndromes in cattle^33–35^, providing further evidence that OvGHV2 is a potential agent of respiratory disease of cattle, as was proposed^2^.

We had previously identified OvGHV2 in association with BRD^34^ and pulmonary disease in cattle^32,33,35^, and in one sheep^37^ with histopathologic evidence of infection but without clinical manifestations of pulmonary disease. Furthermore, in a previous retrospective study of BRD done in Brazil, a MCFV, more likely OvGHV2, was the only pathogen detected in 6.7% (8/120) of cattle with a histological diagnosis of BRD and was the most frequently identified pathogen (53.3%; 64/120) that occurred concomitantly with other common disease agents of pulmonary diseases of cattle, which prompted the theory that this virus may be an adventitious bystander of BRD^2,34^. However, the participation of OvGHV2 in the development of pulmonary disease in ruminants is frequently documented in numerous clinical manifestations of SA-MCF worldwide^2,38–40^. Moreover, the interstitial pneumonia, herein diagnosed, was identified in sheep^41,42^, bison^43^, cattle^44^, and rabbits^45,46^ experimentally infected with OvGHV2. Infected animals had detectable OvGHV2 DNA in the lungs with effect from 1-day post-infection (dpi) through 23 dpi^46^ or 56 dpi^42^, with clinical manifestations of disease occurring at 19 dpi^46^. Therefore, these infectious studies have confirmed that initial replication of OvGHV2 can occur within the lungs^42,45,46^, and provide a plausible explanation for the severe pneumonia identified in the 2-day-old calf during this study. Consequently, the documented experimental^41–46^, and natural^32,34,35,37^ infectious of OvGHV2 associated pulmonary diseases, as well as the results herein described, corroborate with the hypothesis that OvGHV2 should be considered as a potential pathogenic agent of BRD^2^.

The clinical manifestations of nasal and ocular discharge, dyspnea, hyperthermia, and cough observed in the calves with pulmonary discomfort are commonly observed in cattle with BRD^29,30,47^, while the nasal and ocular discharge with hyperthermia are typical clinical manifestations of SA-MCF^2,4,48^. Although the infection was acute in the calf that died with histological evidence of pulmonary lesions, the cow demonstrated the classical manifestations of chronic OvGHV2-related infection due to the PVL identified. Similar arterial lesions were identified in cattle^7,32,49,50^, sheep^37^, and bison^40,51^ infected with OvGHV2. The PVLs identified in this cow can be classified as Grade 3 arterial lesions associated with infection by OvGHV2, being part of the spectrum of arterial disruptions identified in affected animals^40^. These lesions have been identified in cattle^49,50^ and bison^40,51^ that have survived infections by OvGHV2, and should be considered as the consequences (end-result) of initial infections by OvGHV2^50^.

During this study, two asymptomatic calves were infected by OvGHV2. Previous studies have shown that OvGHV2 can infect animals without the demonstration of the typical clinical manifestations of SA-MCF^7,9,32,40,52,53^, suggesting that infections associated with this virus can be subclinical^9,32^ and/or asymptomatic^32^, as occurred in these two animals. Additionally, a retrospective study demonstrated that 42.3% (11/26) of animals infected with OvGHV2 did not have typical clinical signs of SA-MCF^53^. Therefore, the actual incidence of infections due to OvGHV2 may be underestimated^48,49^.

Although the exact trigger mechanism that initiated the acute respiratory disease was not confirmed, the introduction of new farmhands probably contributed to alterations in routine handling practices and may be an indirect contributory factor towards the establishment of stress-related conditions of BRD at this farm^47,54^. Although sheep were not evaluated for the presence of OvGHV2 during this investigation, the age of the lambs at this farm corresponds to the period of major shedding of this virus in asymptomatic sheep^48^. Consequently, the source of dissemination associated with the development of OvGHV2-related infections at this farm probably occurred via contact of susceptible cattle with the virus-laden secretions of asymptomatic sheep during the intermingling of these species, when the enclosed sheep escaped their fenced area, considering that sheep are the reservoir hosts for this virus^3,4^. Additionally, since both sheep and cattle were maintained at the same farm, were only less than 100 m distant and shared the same water and foodstuff, the possibility of infection via the ingestion of contaminated water or feed^48^, cannot be excluded. Furthermore, since the diagnosis of OvGHV2 infection was confirmed, the farmer was notified and has subsequently culled all sheep from his farm.

During this investigation, calves developed an initial respiratory distress syndrome followed by diarrhea two weeks after the onset of respiratory disease. Moreover, antigens and nucleic acids of OvGHV2 were detected within the lungs and intestines of affected cattle, confirming the participation of this virus in the development of the pulmonary and intestinal lesions herein described. However, BVDV1b and Aichivirus were also detected in the fecal samples of some of these calves. While BRV and BCoV were not detected in fecal sample extracts or the intestinal samples of the affected cattle, suggesting that these agents were not associated with the enteric disease observed in the current study. Consequently, it can be argued that the immunodepressive effects of BVDV infections^55^ could have contributed to the development of the disease conditions of these four calves that were simultaneous infected with OvGHV2. However, 12 animals from this study were not infected by BVDV. Therefore, the initial pulmonary infection followed by enteric disease herein described are in accordance with the known pathogenesis of OvGHV2, since initial lytic replication occurs in the lungs^42,45,46,56^ with viral dissemination to other tissues via lymphocytic dissemination occurring at least 2 weeks thereafter^42,45,46^. Furthermore, initial viral replication in the lungs is necessary for the onset of pneumonia and production of progeny viruses that subsequently infects lymphocytes, resulting in systemic dissemination of OvGHV2^45^. Accordingly, most of the calves in the current study that were infected, demonstrated the continuation of the same infectious disease process, rather than the occurrence of the different manifestations of SA-MCF.

Three concomitant respiratory infections were identified during this investigation, all including the participation of OvGHV2, with the simultaneous detection of BoGHV6 (*n* = 2) and BCoV (n = 1) from the DNS of two calves with respiratory distress and one calf with diarrhea. Similar findings of simultaneous agents associated with the development of BRD were described worldwide in dairy^34,57,58^ and feedlot^29,34,59,60^ cattle worldwide, indicating that this is a common phenomenon of cattle with pulmonary disease. Although the possible role of BoGHV6 in the development of diseases in ruminants remains controversial^22^, several studies have associated this pathogen with a wide range of diseases syndromes, including proliferative lesions^20,21^, as wells as pulmonary^17^, intestinal^17^, and reproductive^23,24,26,27^ alterations. Therefore, it is plausible to argue that both *Macavirus*, OvGHV2 and BoGHV6, are agents associated with respiratory disease in ruminants. Although BCoV is associated with enteric disease due to its dual tropism for the respiratory and digestive systems^61^, this virus was only amplified from the DNS of one calf that was simultaneously infected with OvGHV2 without being detected from the intestinal and fecal samples evaluated. Accordingly, these findings confirmed that BCoV was associated only with the development of the pulmonary distress syndrome herein described; similar results were previously described^31,58,60,62,63^.

In conclusion, OvGHV2 was the predominant infectious disease agent identified in dairy cattle that initially developed a pulmonary distress syndrome with clinical manifestations of an enteric disease two weeks after the onset of respiratory discomfort. These findings suggest that OvGHV2 was directly associated with the development of the pulmonary manifestations and indicate that this pathogen should be included within the list of viral agents associated with the development of BRD.

## Methods

### Study location and animals

The study was done at a medium-sized dairy farm located on the outskirts of the city of Francisco Beltrão, in the southwest region of Paraná state, Southern Brazil. The state of Paraná is the second largest (4.6 billion L) producer of milk in Brazil, while in 2020, the region of Francisco Beltrão contributed to an annual production of 89.4 million L and was considered the fourth milk-producing region of Paraná^64^. This farm consisted of 200 lactating dairy cows and 100 calves of the Holstein–Friesian breed of cattle, with an average milk production of 40L/cow/day. Calves at this farm are immunized against BoAHV1, BVDV, BPIV-3, BRSV, *Brucella abortus*, and *Leptospira* spp., and were maintained on a combination of corn silage, hay, and a commercial ration; water was provided ad libitum from artesian wells.

Recently born calves suckle colostrum from their dam at birth until approximately 3 days of age; those that are older received milk (6 L/day) from lactating cows until 21 days of age, at which time they are administered a commercial milk product. Sheep (*n* = 40) were reared at this farm for local consumption and were maintained in a separate area but within proximity to cattle (cows and calves). The sheep population consisted of adults (*n* = 30) and adolescents (*n* = 10). Furthermore, the consulting veterinarian reported that some sheep had escaped their fenced enclosure and intermingled with the cows and calves.

### Clinical data and sampling

The outbreak began in early April 2022, which coincided with the acquisition of recently employed farmhands who were not fully adapted to all managerial aspects of the daily farming routine. The consulting veterinarian indicated that at the time of visit, several cows at this farm had feces that were soft to diarrheic in consistency. Acute respiratory discomfort was observed in 30% (30/100) of the calves, 55% had some form of diarrhea, with both manifestations being observed in several calves, while 9.1% (5/55) of the sick calves died after initial clinical manifestations of respiratory distress. The animals affected were between 1 and 26 months of age. Furthermore, the cases of respiratory discomfort affecting the calves were sporadic, occurring initially during the first and predominantly, the second week after birth; with the affected animals demonstrating nasal and ocular secretions, dyspnea, hyperthermia, and cough. Most of the affected calves initially developed tachypnea with death occurring within 12 to 48 h after the initial onset of clinical manifestations. Additionally, the calves that survived the acute respiratory distress syndrome developed watery diarrhea and were moderately dehydrated. Cows at this farm also developed similar respiratory difficulties resulting in the death of three animals from this category.

Deep nasal swabs (DNS) were collected from calves (*n* = 12) for the molecular detection of pathogens associated with the development of pulmonary and enteric diseases of cattle: 2 were from asymptomatic calves, 3 from calves with pulmonary discomfort, and 7 from calves with manifestations of diarrhea after initial episode of respiratory impairment (Table 1). Additionally, fecal samples (*n* = 11) were obtained from most of the calves from which DNS were collected, except for calves #3, 4, 5 and 7. All samples were maintained on ice and immediately sent for molecular analysis.

Furthermore, *post-mortem* examinations were done on-site by the consulting veterinarian on four animals (3 calves and 1 cow) that died during this outbreak. Pulmonary and intestinal sections from these animals were submitted for molecular evaluation; while intestinal and pulmonary tissue fragments from two of these (# 6 and 8) were fixed by immersion in 10% formalin solution and submitted for routine histopathology.

### Histopathologic, immunohistochemical, and histochemical, evaluations

Fragments of the intestines and lungs of the two animals received for histopathological evaluation were routinely processed with the Hematoxylin and eosin stain. Selected formalin-fixed paraffin-embedded (FFPE) tissue sections from these were used in IHC assays designed to identify tissue antigens of specific agents. These included MCFV^7^, BoAHV1, BRSV, BVDV, and *M. bovis*^34^. Positive controls consisted of FFPE tissue sections known to contain antigens of BoAHV1, BRSV, BVDV, *M. bovis*^34^, and OvHGV2^37^. Two negative controls were used in all IHC assays: the first consisted of replacing the primary antibodies by their respective diluent, while in the second, the primary antibodies were administered on FFPE tissues known to demonstrate negative immunoreactive to the primary antibodies. A list of antibodies, dilutions, methods of antigen retrieval, and source manufactures of the antibodies used in the IHC assays during this study is provided (Supplementary Table 2). Furthermore, selected FFPE tissue sections of the lungs were stained by the Verhoeff-Van Gieson (VVG) histochemical method to identify elastin.

### Molecular detection of infectious disease pathogens of respiratory and enteric diseases of cattle

Nucleic acid extraction was performed from 500 µL proteinase K pre-treated aliquots of tissue suspensions of the DNS, lung, intestines, and fecal samples using a combination of the phenol/chloroform/isoamyl alcohol and silica/guanidine isothiocyanate methods^65,66^. The extracted nucleic acids were eluted in 50 µL of UltraPure DEPC-treated water (Invitrogen Life Technologies, Carlsbad, CA, USA) and stored at -80 °C.

Molecular assays were performed to amplify the nucleic acids of the principal infectious disease agents associated with BRD. These included OvGHV2^67^, BoGHV6^20^, BVDV^68^, BRSV^69^, BoAHV1^70^, BCoV^71^, BPIV-3^72^, *M. haemolytica*^73^, *P. multocida*^74^, *H. somni*^75^, and *M. bovis*^76^. The 11 fecal samples received were evaluated for the detection of infectious agents associated with the development of enteric diseases of ruminants including: BVDV^68^, OvGHV2^67^, BCoV^71^, bovine rotavirus (BRV)^77^, and Aichivirus B^78^. A list of the specific gene targets, primers, and amplicon size obtained during the molecular assays used to identify infectious disease pathogens of respiratory and enteric diseases of cattle is provided (Supplementary Table 3).

Positive controls consisted of the utilization of nucleic acids of these infectious agents derived from previous studies: OvGHV2, BoGHV6^22^, BPIV3, BVDV, BRSV, BoAHV1, BCoV, *M. haemolytica*, *P multocida*, *H. somni*^34,62,79^, BRV, and Aichivirus^80^. Sterile, ultrapure water was used as the negative control in all nucleic acid extractions and procedures.

### Sequencing of OvGHV2, BoGHV6, and BCoV

The products of the molecular assays designed to amplify the nucleic acids of OvGHV2, BoGHV6, and BCoV from the DNS, lung, and intestine of these animals were purified using the PureLink Quick Gel Extraction and PCR Purification Combo Kit (Invitrogen Life Technologies, Carlsbad, CA, USA), quantified by using a Qubit Fluorometer (Invitrogen Life Technologies, Eugene, OR, USA), and submitted to direct sequencing in both directions with the forward and reverse primers used in the respective molecular assays in an ABI3500 Genetic Analyzer sequencer with the BigDye Terminator v3.1 Cycle Sequencing Kit (Applied Biosystems, Foster City, CA, USA).

Sequence quality analyses and consensus sequences were obtained using PHRED and CAP3 software (http://asparagin.cenargen.embrapa.br/phph/), respectively. Similarity searches of the OvGHV2 tegument protein, the BoGHV-6 polymerase, and the BCoV N genes were performed with the respective nucleotide (nt) sequences deposited in GenBank using the Basic Local Alignment Search Tool software (https://blast.ncbi.nlm.nih.gov/Blast.cgi). The identity of the nt sequences was confirmed by comparison with reference sequences available in GenBank.

### Ethics approval

All applicable international, national, and/or institutional guidelines for the care and use of animals were followed. Additionally, permission was obtained from the owner of this animal relative to the utilization in scientific studies. Moreover, permission to realize studies in cattle was obtained from the National Council for the Control of Animals in Experiments (CONCEA; Brazil) and approved by the Animal Ethics Committees for Animal Usage of the Universidade Estadual de Londrina (CEUA/UEL; protocol, 835.2019.45).

### ARRIVE guidelines

All methods are reported in accordance with ARRIVE guidelines.

## Supplementary Information

Supplementary Figure 1.

Supplementary Table 1.

Supplementary Table 2.

Supplementary Table 3.

## Data Availability

The nucleotide sequence of the OvGHV2, BoGHV6, and BCoV strains identified during this study are deposited in GenBank (https://www.ncbi.nlm.nih.gov/genbank/). OvGHV2/BRA/UEL/PR-447/22 and OvGHV2/BRA/UEL/PR-450/22, GenBank accessions (# OP121120 and OP121121, respectively); BoGHV6/BRA/UEL/PR-450/202 (GenBank accession # OP121119) and BCoV/BRA/PR-447-320/2022-N (GenBank accession # OP121122).
