# Supplementary material for: Association of ovine gammaherpesvirus 2 with an outbreak of acute respiratory disease in dairy cattle
Source: Sci Rep. 2023 Apr 6;13:5623. doi: 10.1038/s41598-023-30133-w (PMC10078036; doi:10.1038/s41598-023-30133-w)
Supplement: Supplementary file 2 — Supplementary Table 1. [file 41598_2023_30133_MOESM2_ESM.docx]

Association of ovine gammaherpesvirus 2 with an outbreak of acute

respiratory disease in dairy cattle

Selwyn Arlington Headley,^1,2^ Alais M. Dall Agnol,^3^ José Antonio Bessegato,^4^

Ana Paula Souza Frucchi,^3^ Érika Fernandes Lopes Maturana,^1^ Rafael Vince Rodrigues^1^

Ana Aparecida Correa Xavier,^1,^ Alice Fernandes Alfieri,^2,3^

Amauri Alcindo Alfieri^2,3^

^1^Laboratory of Animal Pathology, Department of Veterinary Preventive Medicine, Universidade Estadual de Londrina, Paraná, Brazil

^2^National Institute of Science and Technology for Dairy Production Chain (INCT – LEITE), Department of Preventive Veterinary Medicine, Universidade Estadual de Londrina, Paraná, Brazil

^3^Laboratory of Animal Virology, Department of Preventive Veterinary Medicine, Universidade Estadual de Londrina, Paraná, Brazil

^4^ Consulting Veterinarian, Herd Bovinos - Consultoria Pecuária, Dois Vizinhos, Paraná, Brazil.

Corresponding author

Dr. Selwyn A. Headley, Laboratory of Animal Pathology, Department of Veterinary Preventive Medicine, Universidade Estadual de Londrina, Paraná, Rodovia Celso Garcia Cid, PR 445 Km 380, Campus Universitário, PO Box 10.011, 86057-970. Brazil. Phone: + 55 43 3371-4766. E-mail: [selwyn.headley@uel.br](mailto:selwyn.headley@uel.br)

Supplementary Table 1. Comparative nucleotide sequence identity of the viruses identified in dairy cattle with respiratory distress.

| **Type of**  **virus** | **Reference**  **strains** | **GenBank**  **accession #** | **Name of identified strain** | **Bp** | **% nt**  **Identity** |
| --- | --- | --- | --- | --- | --- |
| Bovine gammaherpesvirus 6 | Pennsylvania 47 NC024303 | OP121119 | BoGHV6/BRA/UEL/PR-450/2022 | 549 | 100 |
| Bovine coronavirus | Mebus  U00735 | OP121122 | BCoV/BRA/PR-447-320/2022-N | 253 | 99.2 |
| Ovine gammaherpesvirus 2 | BJ1035 NC007646 | OP121120 | OvGHV2/BRA/UEL/PR-447/22 | 237 | 97.8 |
|  |  | OP121121 | OvGHV2/BRA/UEL/PR-450/22 | 237 | 98.7 |
|  |  |  |  |  |  |
