# Supplementary material for: Association of ovine gammaherpesvirus 2 with an outbreak of acute respiratory disease in dairy cattle
Source: Sci Rep. 2023 Apr 6;13:5623. doi: 10.1038/s41598-023-30133-w (PMC10078036; doi:10.1038/s41598-023-30133-w)
Supplement: Supplementary file 3 — Supplementary Table 2. [file 41598_2023_30133_MOESM3_ESM.docx]

Association of ovine gammaherpesvirus 2 with an acute respiratory disease syndrome in dairy cattle

Selwyn Arlington Headley,^1,2^ Alais M. Dall Agnol,^3^ José Antonio Bessegato,^4^

Ana Paula Souza Frucchi,^3^ Érika Fernandes Lopes Maturana,^1^ Rafael Vince Rodrigues^1^

Ana Aparecida Correa Xavier,^1,^ Alice Fernandes Alfieri,^2,3^

Amauri Alcindo Alfieri ^2,3^

^1^Laboratory of Animal Pathology, Department of Veterinary Preventive Medicine, Universidade Estadual de Londrina, Paraná, Brazil

^2^National Institute of Science and Technology for Dairy Production Chain (INCT – LEITE), Department of Preventive Veterinary Medicine, Universidade Estadual de Londrina, Paraná, Brazil

^3^Laboratory of Animal Virology, Department of Preventive Veterinary Medicine, Universidade Estadual de Londrina, Paraná, Brazil

^4^ Consulting Veterinarian, Herd Bovinos - Consultoria Pecuária, Dois Vizinhos, Paraná, Brazil.

Corresponding author

Dr. Selwyn A. Headley, Laboratory of Animal Pathology, Department of Veterinary Preventive Medicine, Universidade Estadual de Londrina, Paraná, Rodovia Celso Garcia Cid, PR 445 Km 380, Campus Universitário, PO Box 10.011, 86057-970. Brazil. Phone: + 55 43 3371-4766. E-mail: [selwyn.headley@uel.br](mailto:selwyn.headley@uel.br)

Supplementary Table 2. List of antibodies, dilutions, method of antigen retrieval, and source manufactures of the immunohistochemical assays.

| **Antibody** | **Clonality** | **Antigen retrieval** | **Dilution** | **Source** |
| --- | --- | --- | --- | --- |
| BoAHV1 (MAb gC-gIII) | Monoclonal | Citrate buffer (pH 6.0) | 1:700 | VRMD, Pullman, WA, USA |
| BPIV-3 | Monoclonal | EDTA buffer (pH 9.0) | 1:40 | Gently ceded by Dr. Eduardo F. Flores, UFSM |
| BRSV (15c7) | Monoclonal | Citrate buffer (pH 6.0) | 1:300 | Gently ceded by Dr. Eduardo F. Flores, UFSM |
| BVDV (13/G4) | Monoclonal | Citrate buffer (pH 6.0) | 1:300 | Santa Cruz Biotechnology, Dallas, Texas, USA |
| *Mycoplasma bovis* | Polyclonal | Citrate buffer (pH 6.0) | 1:10 | Gently ceded by Dr. Lucienne Preto-Giordano, UEL |
| MCFV (MAb-15A) | Polyclonal | Citrate buffer (pH 6.0) | 1:400 | Gently ceded by Dr. Cristina W. Cunha, USDA, Pullman, WA, USA |

Footnote: BoAHV1: bovine alphaherpesvirus 1; BPIV-3: bovine parainfluenza virus 3; BRSV: bovine respiratory syncytial virus; BVDV: bovine viral diarrhea virus type 1; MCFV, malignant catarrhal fever virus.
