# Supplementary material for: Association of ovine gammaherpesvirus 2 with an outbreak of acute respiratory disease in dairy cattle
Source: Sci Rep. 2023 Apr 6;13:5623. doi: 10.1038/s41598-023-30133-w (PMC10078036; doi:10.1038/s41598-023-30133-w)
Supplement: Supplementary file 4 — Supplementary Table 3. [file 41598_2023_30133_MOESM4_ESM.docx]

Association of ovine gammaherpesvirus 2 with an acute respiratory disease syndrome in dairy cattle

Selwyn Arlington Headley,^1,2^ Alais M. Dall Agnol,^3^ José Antonio Bessegato,^4^

Ana Paula Souza Frucchi,^3^ Érika Fernandes Lopes Maturana,^1^ Rafael Vince Rodrigues^1^

Ana Aparecida Correa Xavier,^1,^ Alice Fernandes Alfieri,^2,3^

Amauri Alcindo Alfieri ^2,3^

^1^Laboratory of Animal Pathology, Department of Veterinary Preventive Medicine, Universidade Estadual de Londrina, Paraná, Brazil

^2^National Institute of Science and Technology for Dairy Production Chain (INCT – LEITE), Department of Preventive Veterinary Medicine, Universidade Estadual de Londrina, Paraná, Brazil

^3^Laboratory of Animal Virology, Department of Preventive Veterinary Medicine, Universidade Estadual de Londrina, Paraná, Brazil

^4^ Consulting Veterinarian, Herd Bovinos - Consultoria Pecuária, Dois Vizinhos, Paraná, Brazil.

Corresponding author

Dr. Selwyn A. Headley, Laboratory of Animal Pathology, Department of Veterinary Preventive Medicine, Universidade Estadual de Londrina, Paraná, Rodovia Celso Garcia Cid, PR 445 Km 380, Campus Universitário, PO Box 10.011, 86057-970. Brazil. Phone: + 55 43 3371-4766. E-mail: [selwyn.headley@uel.br](mailto:selwyn.headley@uel.br)

Supplementary Table 3. Targets genes, primers, and amplicon size of the molecular assays used to identify infectious disease pathogens of respiratory and enteric diseases of cattle.

| **Pathogens** | **Target genes** | **Primer sequencies (5´ - 3´)** | **Amplicon size (bp)** | **Reference**  **order** |
| --- | --- | --- | --- | --- |
| **Viral** | | | | |
| OvGHV2 | Tegument protein | Fw-AGTCTGGGTATATGAATCCAGATGGCTCTC | 422 | ^1^ |
|  |  | Rv-AAGATAAGCACCAGTTATGCATCTGATAAA |  |  |
| BoGHV6 | DNA polymerase | Fw-ACAGACGGGCAGCAGATAAG | 551 | ^2^ |
|  |  | Rv-ATGGTTCGCCCCTGTAGAGT |  |  |
| BCoV | N gene | Fw-TTGCTAGTCTTGTTCTGGC | 251 | ^3^ |
|  |  | Rv-TGTGGGTGCGAGTTCTGC |  |  |
| BVDV | 5'UTR | Fw-ATGCCC(T/A)TAGTAGGACTAGCA | 288 | ^4^ |
|  |  | Rv-TCAACTCCATGTGCCATGTAC |  |  |
| BRSV | G gene | Fw-CATCAATCCAAAGCACCACACTGTC | 371 | ^5^ |
|  |  | Rv-GCTAGTTCTGTGGTGGATTGTTGTC |  |  |
| BoAHV1 | C gene | Fw-CAACCGAGACGGAAAGCTCC | 354 | ^6^ |
|  |  | Rv-AGTGCACGTACAGCGGCTCG |  |  |
| BPIV-3 | HN gene | Fw-GAATGACTCATGATAGAGGTAT | 647 | ^7^ |
|  |  | Rv-AGGACAACCAGTTGTATTACAT |  |  |
| BRV | VP7 | Fw-GGCTTTAAAAGAGAGAATTTCCGTCTGG | 1062 | ^8^ |
|  |  | Rv-GGTCACATCATACAATTCTAATCTAAG |  |  |
| Aichivirus B | 3D | Fw-TGGAYTACAAG(/R)TGTTTTGATGC | 216 | ^9^ |
|  |  | Rv-ATGTTGTTRATGATGGTGTTGA |  |  |
| **Bacterial** | | | | |
| *Mannheimia* *haemolytica* | lktA-artJ intergenic region | Fw-GTCCCTGTGTTTTCATTATAAG | 385 | ^10^ |
|  |  | Rv-CACTCGATAATTATTCTAAATTAG |  |  |
| *Histophilus* *somni* | 16S | Fw-GAAGGCGATTAGTTTAAGAG | 408 | ^11^ |
|  |  | Rv-TTCGGGCACCAAGTRTTCA |  |  |
| *Pasteurella multocida* | ORF KMT1 | Fw-GCTGTAAACGAACTCGCCAC | 460 | ^12^ |
|  |  | Rv-ATCCGCTATTTACCCAGTGG |  |  |
| *Mycoplasma bovis* | 16S-23S rRNA internal transcribed spacer | Fw-GTACACTTGTCTTTTATCACTATA | 488 | ^13^ |
|  |  | Rv-AAGGTATCTCGCTTTATGTCCT |  |  |

**References**

1 Baxter, S. I., Pow, I., Bridgen, A. & Reid, H. W. PCR detection of the sheep-associated agent of malignant catarrhal fever. *Arch Virol* **132**, 145-159 (1993). <https://doi.org:10.1007/bf01309849>

2 Oliveira, C. H. *et al.* Bovine herpesvirus 6 in buffaloes (*Bubalus bulalis*) from the Amazon region, Brazil. *Trop Anim Health Prod* **47**, 465-468 (2015). <https://doi.org:10.1007/s11250-014-0733-z>

3 Takiuchi, E., Stipp, D. T., Alfieri, A. F. & Alfieri, A. A. Improved detection of bovine coronavirus N gene in faeces of calves infected naturally by a semi-nested PCR assay and an internal control. *J Virol Methods* **131**, 148-154 (2006). <https://doi.org:10.1016/j.jviromet.2005.08.005>

4 Vilcek, S. *et al.* Pestiviruses isolated from pigs, cattle and sheep can be allocated into at least three genogroups using polymerase chain reaction and restriction endonuclease analysis. *Arch Virol* **136**, 309-323 (1994). <https://doi.org:10.1007/bf01321060>

5 Vilcek, S., Elvander, M., Ballagi-Pordany, A. & Belak, S. Development of nested PCR assays for detection of bovine respiratory syncytial virus in clinical samples. *J Clin Microbiol* **32**, 2225-2231 (1994).

6 Claus, M. P. *et al.* Rapid detection and differentiation of bovine herpesvirus 1 and 5 glycoprotein C gene in clinical specimens by multiplex-PCR. *J Virol Methods* **128**, 183-188 (2005). <https://doi.org:10.1016/j.jviromet.2005.05.001>

7 Zhu, Y. M. *et al.* Isolation and genetic characterization of bovine parainfluenza virus type 3 from cattle in China. *Vet Microbiol* **149**, 446-451 (2011). <https://doi.org:10.1016/j.vetmic.2010.11.011>

8 Gouvea, V. *et al.* Polymerase chain reaction amplification and typing of rotavirus nucleic acid from stool specimens. *J Clin Microbiol* **28**, 276-282 (1990).

9 Reuter, G., Boldizsár, A. & Pankovics, P. Complete nucleotide and amino acid sequences and genetic organization of porcine kobuvirus, a member of a new species in the genus Kobuvirus, family Picornaviridae. *Arch Virol* **154**, 101-108 (2009). <https://doi.org:10.1007/s00705-008-0288-2>

10 Angen, O. *et al.* Respiratory disease in calves: microbiological investigations on trans-tracheally aspirated bronchoalveolar fluid and acute phase protein response. *Vet Microbiol* **137**, 165-171 (2009). <https://doi.org:10.1016/j.vetmic.2008.12.024>

11 Angen, O., Ahrens, P. & Tegtmeier, C. Development of a PCR test for identification of *Haemophilus somnus* in pure and mixed cultures. *Vet Microbiol* **63**, 39-48 (1998).

12 Townsend, K. M., Frost, A. J., Lee, C. W., Papadimitriou, J. M. & Dawkins, H. J. Development of PCR assays for species- and type-specific identification of *Pasteurella multocida* isolates. *J Clin Microbiol* **36**, 1096-1100 (1998). <https://doi.org:10.1128/jcm.36.4.1096-1100.1998>

13 Voltarelli, D. C. *et al.* A nested-PCR strategy for molecular diagnosis of mollicutes in uncultured biological samples from cows with vulvovaginitis. *Anim Reprod Sci* **188**, 137-143 (2018). <https://doi.org:10.1016/j.anireprosci.2017.11.018>
